# Multidrug-Resistant TB (MDR-TB) and Extensively Drug-Resistant TB (XDR-TB) Among Children: Where We Stand Now

**DOI:** 10.7759/cureus.35154

**Published:** 2023-02-18

**Authors:** Kona Chowdhury, Rahnuma Ahmad, Susmita Sinha, Siddhartha Dutta, Mainul Haque

**Affiliations:** 1 Pediatrics, Gonoshasthaya Samaj Vittik Medical College, Dhaka, BGD; 2 Physiology, Medical College for Women and Hospital, Dhaka, BGD; 3 Physiology, Khulna City Medical College, Khulna, BGD; 4 Pharmacology, All India Institute of Medical Sciences, Rajkot, IND; 5 Pharmacology and Therapeutics, National Defence University of Malaysia, Kuala Lumpur, MYS

**Keywords:** sustainable development goals (sdgs), target regarding tb management, who- world health organization, new financial investment., treatment success, pediatric population, mycobacterium tuberculosis, extensively drug-resistant tb (xdr tb), multidrug-resistant tb (mdr tb), tuberculosis

## Abstract

Drug-resistant tuberculosis (DR-TB) has continued to be a global health cataclysm. It is an arduous condition to tackle but is curable with the proper choice of drug and adherence to the drug therapy. WHO has introduced newer drugs with all-oral shorter regimens, but the COVID-19 pandemic has disrupted the achievements and raised the severity. The COVID-19 controlling mechanism is based on social distancing, using face masks, personal protective equipment, medical glove, head shoe cover, face shield, goggles, hand hygiene, and many more. Around the globe, national and international health authorities impose lockdown and movement control orders to ensure social distancing and prevent transmission of COVID-19 infection. Therefore, WHO proposed a TB control program impaired during a pandemic. Children, the most vulnerable group, suffer more from the drug-resistant form and act as the storehouse of future fatal cases. It has dire effects on physical health and hampers their mental health and academic career. Treatment of drug-resistant cases has more success stories in children than adults, but enrollment for treatment has been persistently low in this age group. Despite that, drug-resistant childhood tuberculosis has been neglected, and proper surveillance has not yet been achieved. Insufficient reporting, lack of appropriate screening tools for children, less accessibility to the treatment facility, inadequate awareness, and reduced funding for TB have worsened the situation. All these have resulted in jeopardizing our dream to terminate this deadly condition. So, it is high time to focus on this issue to achieve our Sustainable Development Goals (SDGs), the goal of ending TB by 2030, as planned by WHO. This review explores childhood TB's current position and areas to improve. This review utilized electronic-based data searched through PubMed, Google Scholar, Google Search Engine, Science Direct, and Embase.

## Introduction and background

The theme of World tuberculosis (TB) day of the year 2022 was - "Invest to end TB. Save lives [1]." This message reflects the utmost importance of fighting against TB, one of the topmost infectious killers in the world [2,3]. It has been appraised that 997500 incidence TB cases among pediatric folk. Among them, 481000 and 516500 cases were 0-4-years and 5-14-years old, respectively, in 2019 [4]. Another study reported a shortfall of comprehensive data on epidemiology and DR-TB portrayal among childhood TB cases [5]. One more investigation revealed that laboratory MDR-TB global prevalence was 3.2% among the pediatric group [6]. The Coronavirus disease (COVID-19) pandemic has reversed the progress achieved by several TB control programs worldwide, and the fight against TB has been set back by several years [7,8].

Moreover, due to evolving ability of Mycobacterium tuberculosis against anti-TB drugs [9], drug-resistant TB cases are on the rise, which is quite apparent [10,11]. Adult drug-resistant cases are the source of infection in children [12], who become easy prey and gradually develop severe progressive TB [13,14]. The low availability of diagnostic tests [15] and the time-consuming, costly treatment [16,17] are the main hindrances to successful treatment. Moreover, interactions between drugs [18,19], their side effects [20-22], and consequently, therapeutic failure [23] add misery and burden socially and financially to the patients. So, it is high time to move forward with increased efforts and investments to end TB, including DR-TB.

This narrative review was conducted to investigate the present status of childhood DR-TB and areas to intervene in and, thereby, our future generation have better health and healthcare.

## Review

A brief history of tuberculosis

Tuberculosis is a highly contagious, microbial infective disorder resulting from Mycobacterium tuberculosis (MT) and humankind's earliest known infective illness. TB in humans, perchance, was tracked down as earliest as about 9250 to 8160 (estimated) years ago in a city within the Mediterranean Sea named Atlit Yam [24-27]. This geographical area is situated near the coast of Israel [24]. Paleontologists and excavators detected TB in the skeletal remaining of the mother and offspring interred simultaneously [28]. The only written recorded evidence of TB in the People's Republic of China [29], the Arab Republic of Egypt [30-32], and the Republic of India [30], and were about 2300, 2400, and 3300 years ago, respectively [29-33].

**Figure 1:**
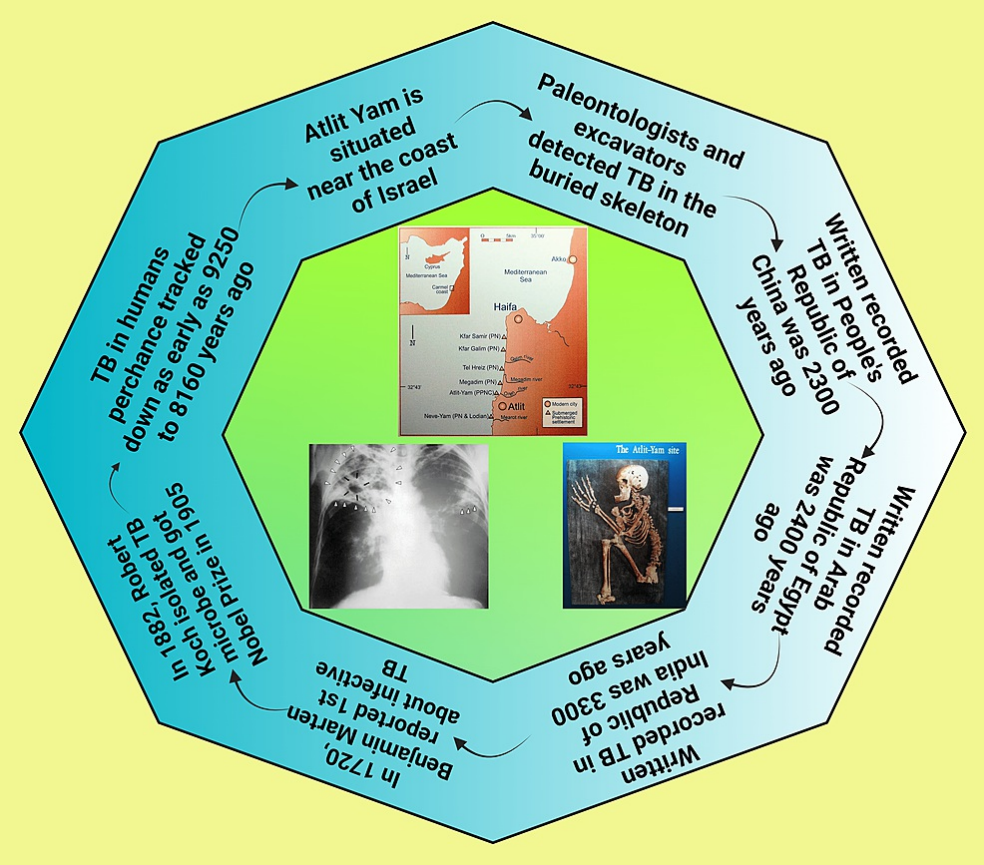
Highlighted events in TB history. Note: This figure has been developed by utilizing the premium version of BioRender (https://biorender.com/) with License No.: CH24W5U6GS. A detail of the history of TB can obtain at the following link http://www.israelandyou.com/atlit-yam/ Image Credit: Susmita Sinha.

A British physician, Benjamin Marten, reported the infective origin of TB, in 1720, in his paper - a new theory of consumption [34]. Later, celebrated German physician-scientist and microbiologist Heinrich Hermann Robert Koch successfully isolated a microbe that caused TB in 1882. One year later, the causative microbe was named Mycobacterium tuberculosis [35,36]. Dr. Robert Koch was awarded the Nobel Prize for his discovery of the causative agent of TB in 1905 [37]. Figure 1 illustrates briefly breaking updated on the history of TB.

Discovery of anti-tuberculosis agents

Para-amino salicylic acid (PAS) and streptomycin were the first antimicrobials in the market that possess adequate anti-tubercular efficacy to combat TB and those prescribed in combination [38]. Subsequently, pyrazinamide, isoniazid, ethionamide, cycloserine, rifampicin, and ethambutol were quickly discovered for TB [39-41]. The rapid development of these anti-tubercular medications has managed and treated TB from a fatal infectious disease to a curable infective disorder [39,42], thereby reducing fatal clinical outcomes from 27% to 7% [19]. However, TB was once thought to be a completely curable disease [43]. Still, with the advent of drug resistance TB, currently available anti-tubercular agents sometimes have failed to combat MT in some extremely resistant cases [9,44,45]. Thereby, globally, TB reappeared as a major killer infectious disease [46,47]. Furthermore, in the last 50 years, only a few anti-tubercular medications such as Bedaquiline (BDQ), delamanid, PA-824 (a bicyclic derivative of nitroimidazole), SQ-109 (a synthetic analog of ethambutol) and benzothiazinones [1,3-benzothiazin-4-one or benzothiazinone (BTZ)] were added in anti- TB medication family that have added worries to combat the morbid infective condition [38,48-53].

Types of drug-resistant tuberculosis and implications

According to WHO, there are 05 types of DR-TB. Those are I. Mono-resistance: resistance to 01 first-line anti-TB medicine at most. II. Poly-resistance: resistance over one first-line anti-TB agent, except for doublet isoniazid and rifampicin. III. Multidrug resistance (MDR): at the minimum, resistance to rifampicin and isoniazid as duplet. IV. Extensive drug resistance (XDR): resistance to any member fluoroquinolone family and no less than 01 of 03 second-line parenteral medications. Those are capreomycin, kanamycin, and amikacin, in addition, to MDR. V. Rifampicin resistance (RR): resistance to rifampicin ascertained by utilizing phenotypic or genotypic modus operands, whether or not resistant to anti-TB agents. It comprises every resistance to rifampicin in mono-resistance, poly-resistance, MDR, or XDR [54]. Another research reported that each year Mycobacterium tuberculosis-infected more than half a million new cases of RR-TB and MDR-TB with resistance to isoniazid and rifampicin [6]. Despite the enormous development in medical sciences, present-day DR-TB carry on as an international health combination and is explained by sky-scarping morbidity and mortality, poorer clinical outcome, high expense, and raised complicated clinical scenario for appropriate pharmacological intervention [55,56].

Furthermore, inadequate and ineffectual application of anti-tubercular medicine manages a considerable fraction of TB cases to remain alive with many morbidities; nonetheless, they left out as carriers of DR-TB and strengthened resistance during the therapeutic intervention and build up and encourage transmission process of TB [57]. However, there is enormous progress regarding interpreting the pathogenesis of TB and the diversity of molecular biology of DR-TB [11,58]. Additionally, evidence suggests that there has been substantial progress in upcoming novel medications and combinations of anti-TB agents [59-61]. However, it has been reported that the first-line anti-TB agents frequently appeared less efficacious globally [59,62]. Moreover, a big gap exists between patients who urgently require second-line medication and those who eventually receive or have access to appropriate pharmacological intervention [59,61]. Despite all problematic issues regarding DR-TB around the globe, it was estimated in 2018 that 1.56 million TB cases having MDR-TB or RR-TB had access to appropriate anti-TB agents. Nevertheless, at most, 56% accomplished treatment auspiciously [6].

Multidrug-resistant tuberculosis and extensively drug-resistant TB

The MDR-TB is restricted to TB, which manifests resistance to isoniazid and rifampicin, the most effective TB medicines [63]. Globally, around 3.4% and 20% of the new TB patients with a history of preceding medication for TB, respectively, were identified with MDR-TB [63]. Another research revealed that those TB cases that received anti-tubercular medication have fourteen folds elevated possibilities of evolving to MDR-TB [52]. It has been reported that in 2018 around 5,00,000 new cases of TB were detected. Of these cases, 78% were MDR-TB [64,65].

**Figure 2:**
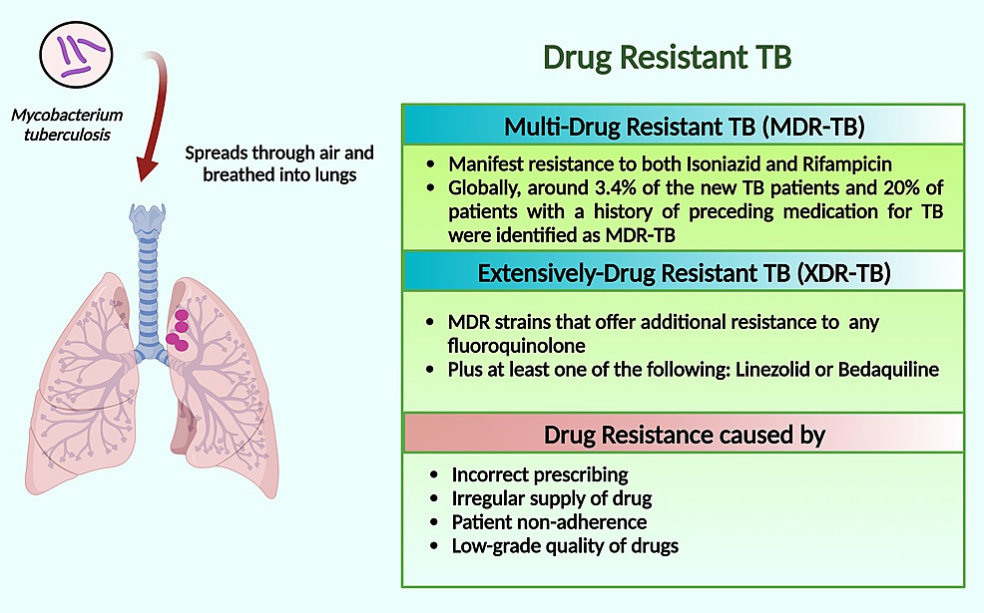
Schematic presentation of multidrug-resistant TB and extensively drug-resistant TB. Note: This figure has been developed by utilizing the premium version of BioRender (https://biorender.com/) with License No.: YJ24X0TAQK. Image Credit: Susmita Sinha.

Two more categories are used by WHO; one is pre-extensively drug-resistant (pre-XDR), shows insusceptibility to rifampicin and one of the fluoroquinolones; another is extensively drug-resistant TB (XDR-TB) which denotes MDR strains that offer additional resistance to any fluoroquinolone plus at least one of the followings - linezolid or bedaquiline (Figure 2) [66]. If there is further resistance beyond XDR, the patient is considered drug-resistant and challenging to treat, ultimately emerging as a deadly nature [67]. This untreatable form of TB currently displays obstacles when there is no effective medicine against TB. The inability to cure the disease results in inflation of the death rate [68,69] and the necessity for other possible practices to counter infection transmission [70].

Medical personnel is particularly at risk of DR-TB because of their work profile and greater chances of encountering patients. It is related to increased morbidity and mortality and expensive treatment resulting in serious health issues and worsening public health problems [71]. "Drug susceptibility testing and deoxyribonucleic acid (DNA) fingerprinting" have given an idea of that DRTB among pediatric community consequences principally transferal of resistant variety of Mycobacterium tuberculosis and by the accession of resistance by way of sparse therapeutic intervention [72,73].

Challenges in diagnosis and treatment in the pediatric age group

Diagnosing DR-TB cannot be done with conventional methods but need modern drug sensitivity testing to establish the drug resistance pattern of the MT. The absence of effective and affordable rapid diagnostic techniques makes the diagnosis arduous [59,74,75]. Several phenotypic and molecular approaches have been explored to develop quick, reliable, and accurate methods for rapidly detecting drug resistance [66]. WHO recommends Cepheid Xpert MTB/RI, also known as GeneXpert, as the initial molecular diagnostic assay for drug resistance detection. This procedure detects the presence of MTB bacilli as well as rifampicin resistance. Due to the sub-optimal sensitivity of Xpert MTB/RIF, Xpert Ultra has been developed to overcome this limitation. In the 2021 WHO guideline, Xpert/MTB RIF has been advised to be used on the specimen from gastric lavage, sputum, nasopharyngeal aspirate, and stool. Xpert Ultra can be used on nasopharyngeal aspirate and sputum [76].

Young children often fail to produce sputum samples. Even if they do so, the number of bacilli is not adequate for bacteriological confirmation [15,77]; as a result, diagnosis is often made clinically, especially when there is a history of contact with a DR-TB case [78,79]. Eventually, this causes starting of treatment empirically [80-82]. Moreover, the culture of TB bacilli, the contemporary gold standard diagnostic method [83,84], requires several weeks to months [85]. Therefore, treatment is initiated chiefly empirically [86], delaying appropriate treatment. This empirical medication frequently causes excessive drug prescribing with unwanted side effects [87], and ineffective treatment raises further resistance [88,89].

**Figure 3:**
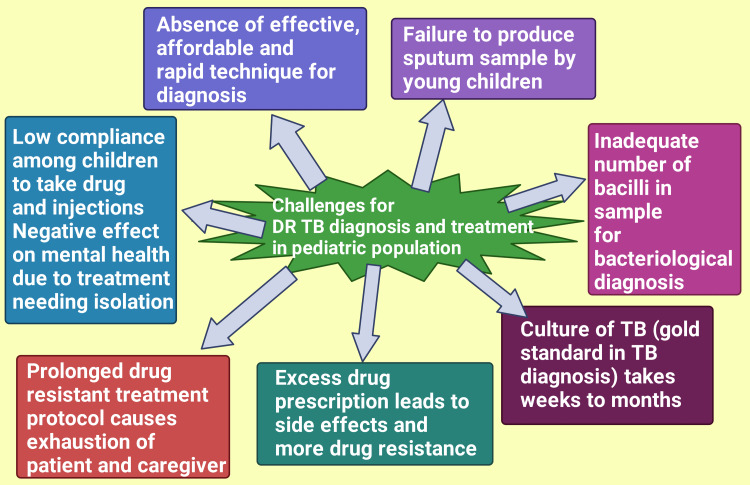
Depicting the various challenges for diagnosing and treating Drug-Resistant Tuberculosis among the pediatric population. Notes: DR-TB: Drug-Resistant Tuberculosis. Note: This figure has been developed by utilizing the premium version of BioRender (https://biorender.com/) with License No.: BV24WGROUB. Image Credit: Rahnuma Ahmad

Treatment of DR-TB in the pediatric age group is more rewarding than in adults but still poses a challenge [90-92]. It has been reported that children often find it burdensome to take anti-TB tablets and painful injections [90,93]. Older children and adolescents are segregated from society, disrupting their self-confidence and hampering academic careers [90,94,95]. Eventually, they face mental health challenges. Drug resistance is often diagnosed in TB cases not cured with first-line medicines [96]. Therefore, the more extended drug-resistant TB treatment protocol, along with a past episode of TB, often causes exhaustion to patients and caregivers [90,97] (Figure 3).

Current Status of Drug-Resistant TB in children

According to WHO, in 2021, an estimated 10.6 million people were affected by TB globally, which was 4.5% more than in 2020. Pulmonary TB was diagnosed in 5.3 million, and bacteriological confirmation was possible in 63% of cases (59% in 2020) [66]. As expected, the confirmation rate was lowest in low-income countries due to insufficient access to diagnostic tests. Seventy-one percent of people from bacteriologically confirmed lung TB cases were found to be rifampicin-resistant. About 141953 and 25038 patients belonged to MDR/RR and pre-XDR TB/ XDR TB groups, respectively. In 2020 this was 6.4% lower. It has added some relief that the enrollment for treatment in drug-resistant cases has increased (7.5% higher) compared to 2020, although it is still lower than in 2019. Unfortunately, the registration of children was low in number. Fewer enrollments have jeopardized our global targets for ending TB, which is already beyond our reach [66,98]. From 2018 to 2021, the percentage of enrollment was only 43% of our five-year target (2018-2022); in drug-resistant pediatric cases, it was only 15% [66].

**Figure 4:**
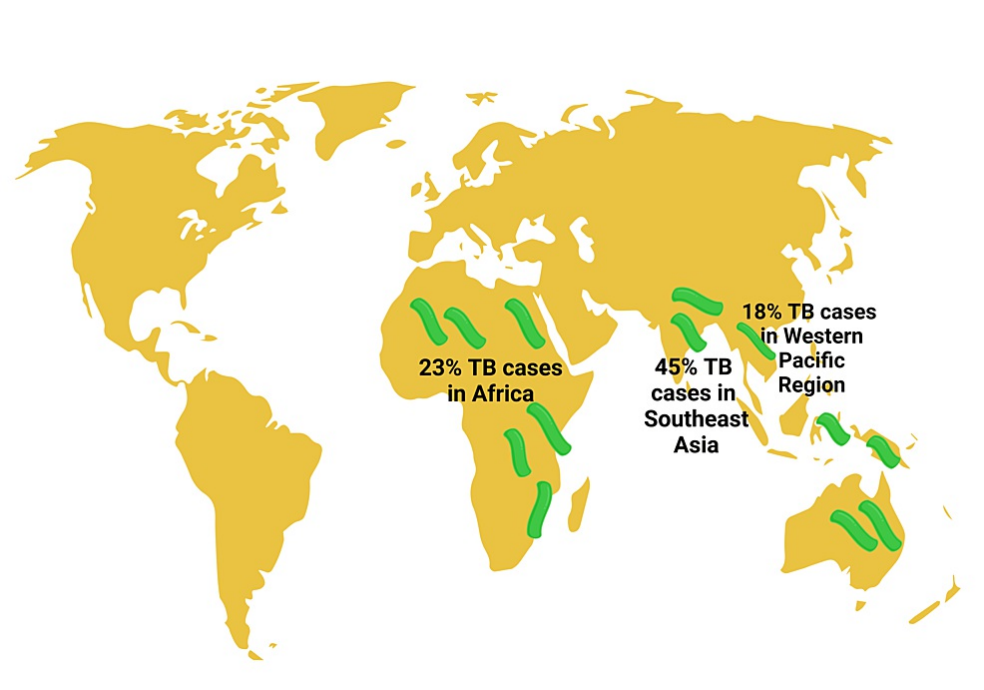
Illustrating the percentage of cases of Tuberculosis in Southeast Asia, Africa, and the Western Pacific region of the earth. Notes: TB: Tuberculosis. Note: This figure has been developed by utilizing the premium version of BioRender (https://biorender.com/) with License No.: ZV24WK8LWK. Image Credit: Rahnuma Ahmad

The highest contribution of global TB cases came from South East Asia (45%), followed by Africa (23%) and the Western Pacific (18%) (Figure 4). Two-thirds of these cases belong to China, Indonesia, India, Nigeria, Philippines, Pakistan, Bangladesh Democratic Republic of Congo, and Bangladesh. Men were affected more, and children contributed 11% of total TB incidences. In 2021, the estimated MDR/RR case was 3.6% among new patients and 18% among previously treated subjects [66]. It is less than that of 2015 [66]. India, Pakistan, and the Russian Federation contributed the most to MDR/RR TB cases worldwide [66]. More countries have now initiated continuous surveillance systems [66]. This system uses rapid molecular tools to test drug resistance [99]. Thirty-eight countries with higher caseloads are keeping records on drug susceptibility in 2021-2025 [66], but there is a significant gap in the drug resistance profiles in childhood TB [98,100,101]. It is estimated that 3% of childhood TB cases become resistant, amounting to 25,000 to 32,000 yearly [102]. Diagnosis and treatment are received by only 3-4% of them, and around 21% of them face death [102].

The treatment success rate for DR-TB has shown us some rays of hope [57,98]. The therapeutic success rate increased to 60% in 2019, compared to 59% and 58% in 2018 and 2017, respectively [66,103,104]. The global success rate in managing DR-TB among the pediatric population is still below the WHO target [54]. Only 38% of children who were less than 14 years received treatment. The lowest rate was observed in Western Pacific Region (25%), followed by South East Asia (35%) [105]. Thereby, increased participation in the management of pediatric TB requires more national and international attention with additional financial support [106,107]. Despite inadequate coverage, response to treatment is far better with the first-line anti-TB regimen (88%) in 2020, which was highest in Southeast Asia. Effective and comparatively low-risk medicines have been incorporated into the regimen [59,108]. All these data of success cannot be applied for childhood DR-TB as the case burden is unknown [109,110], cases are underreported [100,111-113], and proper diagnosis is difficult for kids [114-116]. Suitable pediatric drug preparations are also unavailable [102,117]. Recently Bedaquiline has been recommended for children who are 6 years or more, and Delamanid is allowed in the regimen of children aged 3 years or more [66,118-121], making it an all-oral shorter regimen with fewer adverse side effects [122,123]. Ninety-two countries used all oral shorter regimens in 2021 [66].

A brief delineation of pediatric MDR-TB and XDR-TB

About 3% of the pediatric population with TB have MDR-TB, representing between 25,000-32,000 cases evolve MDR-TB disease annually. Hardly 3-4% of these childhood MDR-TB patients are diagnosed, and access to treatment and, proportionally, 21% of cases passed away [124]. The XDR-TB was coined in 2006 to depict strains of MDR-TB resistant to fluoroquinolones and second-line parenteral anti-tubercular medicine. It has been appraised that 9.6% of MDR-TB cases globally have XDR-TB [81,125]. The therapeutic intervention regarding MDR-TB and XDR-TB among pediatric cases remains with the same principles and uses the same second-line agents for adults [126,127]. However, the ideal dosage schedule of anti-tubercular medication in these cases is still undetermined. After that, MDR-TB frequently faces poorer clinical outcomes, and often these children encounter higher death rates when compared with drug-sensitive TB [127,128]. Another study reported that each patient treatment cost of XDR-TB was US$26,392. It is four folds higher than MDR-TB (US$6772) and 103 folds faster when compared with drug-sensitive TB (US$257) [129]. Efficacious pharmacological intervention for MDR-TB requires 5-7 barely adequate, high-priced, second-line, and third-line medicine for ≥24 months [130]. Frequently these anti-tubercular agents possess a high level of adverse drug reaction. Thereby, this imposes a substantial financial burden both for individuals and for the community [130]. Consequently, patients with MDR-TB and XDR-TB in low and middle-income countries (LMICs) encounter a challenging situation. XDR-TB broadly remains an irremediable disease in LMICs [130]. The research expects a certain amount of available anti-tubercular agents and multiple upcoming agents with novel mechanisms of action in different phases of the drug development, bringing hope to minimize the duration of dosage schedule for anti-TB agent-susceptible TB and hopefully bring better clinical outcomes for MDR-TB and XDR-TB sufferers [130].

Prevention of DR-TB in children

Prevention of DR-TB is one of the main focuses in addressing the deadly situation [131], including preventive therapy and vaccination for children [132,133]. As per WHO, no satisfactory progress has been observed in TB preventive treatment in children under five years since 2019. They were honored to achieve only 40% of our 5-year sub-target on preventive therapy in children [66,134]. It is of utter importance that adult household contacts of TB-affected people should receive preventive therapy when they develop latent infection so that they do not progress to active disease [135,136] and act as the source of infection for children [137]. Only seven hundred thousand people with latent TB infection received TB preventive medication in 2021, thereby jeopardizing our children's future [66]. Moreover, the COVID-19 pandemic set us off track in the BCG (Bacillus Calmette-Guérin) vaccination program, which increased child mortality [138,139]. The coverage was reduced to 84% (4% less than in 2019) in 2021 globally [66].

Management of childhood drug-resistant TB

Resistant to rifampicin only, also known as mono-resistant TB among the pediatric community, are advised to treat with isoniazid, ethambutol, and a fluoroquinolone for a minimum of 1 to 1½ years. Furthermore, it has been recommended to add pyrazinamide for at least the first 2 months [140]. Monodrug-resistant TB is defined as Mycobacterium tuberculosis microbes exhibiting resistance to a single anti-TB drug considered a first-line agent, e.g., isoniazid, rifampin, ethambutol, or pyrazinamide [9]. Although it has been reported that poly-resistant TB (PDR-TB) is rare among the pediatric community [141], nevertheless globally, 1.61% of children suffer from PDR-TB [5]. PDR-TB has been defined as resistance to two or more first-line anti-TB drugs, nonetheless, not to both isoniazid and rifampicin concurrently [141,142]. The WHO has recommended that the PDR-TB treatment regimen be designed with appropriate experience in managing TB cases. Additionally, the particular center must possess the required skill to handle MDR-RB. It is also suggested that a panel of physicians should meet regularly to address the progress of individual cases [143]. MDR-TB among pediatric faction is recommended to be prescribed similar medication to adult cases [140,144]. The findings of four important papers are depicted in Table 1 [145-148]. We have summarized observations of four systematic reviews regarding diagnosing childhood-TB cases in Table 2, indexed in PubMed and published in 2022 [149-152].

**Table 1:** The Key Findings of Four Important Papers. DR-TB: Drug-resistant tuberculosis; MDR-TB: Multidrug-resistant tuberculosis

Authors Name	Journal Details	Background	Result	Conclusion
Kassa-Kelembho et al. [145]	Int J Tuberc Lung Dis. 2004;8(5):574-8	DR-TB is a substantial obstacle to the complete cure of patients. DR TB in children is an indicator of ongoing transmission of this condition in society, but fewer studies have been conducted concerning adult cases.	0.6% of childhood TB cases were found to be MDR.	MDR-TB in children is persisting at the same rate as in adults in Bangui, Central African Republic
Cuevas et al. [146]	J Infect Dis. 2012;205 Suppl 2(Suppl 2): S209-15.	Varied clinical features, lack of appropriate investigative methods, and failure to acquire proper specimens are significant barriers to confirmation of pediatric TB cases, ultimately leading to underreporting.	An integrated standard guideline should be available, with explicit instruction on clinically defining a case of childhood TB and how to confirm it followed by proper reporting.	Standard protocols must be distributed and accepted universally to perceive their effect.
Jenkins et al. [147]	Lancet. 2014;383(9928):1572-9.	Underreporting MDR-TB cases in children is a significant concern that has caused negligence in addressing the issue. To ensure a proper and complete cure, it is necessary to estimate the caseload and understand the diversification between various regions of the world,	The risk of MDR-TB in youngsters and adults is almost equal. Almost 32,000 children were affected by MDR-resistant TB bacilli in 2010.	A large gap was observed in diagnosed and undiagnosed childhood TB cases
Ködmön et al. [148]	Euro Surveill. 2017;22(47):17-00103.	Due to the paucibacillary nature of TB in children, along with difficulty obtaining an appropriate sample, diagnosis of pediatric TB remains challenging. The lack of epidemiological data adds more misery.	Between the year from 2007 to 2015, 18,826 children were affected by TB in the European Economic region and European Union, and among them, 2.7% were multi-drug resistant.	Despite decreased caseload in Europe, diagnostic confirmation needs to be improved as it may hinder the accurate picture of the case burden.

**Table 2:** Outline of Top Four Systematic Childhood Tuberculosis Diagnosis Published within One Year Indexed in Pubmed.

Authors Name	Journal Details	Background	Result	Conclusion
Olbrich et al. [149]	BMJ Paediatr Open. 2022;6(1): e001447.	Pediatric TB remains underdiagnosed. The innovative lateral flow FujiLAM assay detects lipoarabinomannan (LAM) in urine. Nevertheless, pieces of evidence regarding childhood TB continue to persist.	FujiLAM, sensitivity remains between 42-63%.	This study reported the greater precision of FujiLAM. Thereby giving an idea of the method’s potential concerning point-of-care (POC) for detecting childhood TB.
Seid et al. [150]	IJID Reg. 2022; 4:97-104.	TB is a worldwide public health concern. Additionally, apprehension rises skyrockets because of the unavailability of requisite and precise point-of-care identifying procedures. Additionally, getting proper sputum from the base of the pulmonary tree in pediatric cases is complicated.	The sensitivity and specificity of the Mycobacterium tuberculosis enzyme-linked immunosorbent assay (MTB-LAM-ELISA), Alere Determine TB LAM Ag (Alere LAM) test, and the Fuji LAM diagnostic procedure among pediatric cases below 15 years with TB were 16.0% and 95.61%; 45.90% and 80.42%; and 52.32% and 89.37%, respectively.	This study revealed that the Fuji LAM and Alere LAM diagnostic procedure are applicable in diagnosing pediatric TB cases.
Kay et al. [151]	Cochrane Database Syst Rev. 2022; 9(9):CD013359	It has been appraised that around 1000000 pediatric communities are affected by TB. Among them, more or less 226,000 passed away yearly. WHO endorses Xpert MTB/RIF Ultra (Xpert Ultra) as a rapid diagnostic test to diagnose complicated Mycobacterium tuberculosis and rifampicin resistance.	This paper revealed Xpert Ultra's sensitivity to various specimen types. Sputum possesses the highest sensitivity, which goes after gastric aspirate and stool. The nasopharyngeal aspirate owns the least.	This study concluded that although the result varies; nevertheless, Xpert Ultra meticulousness is high among pediatric TB.
Kazi et al. [152]	J Glob Health. 2022; 12:10010.	Pediatric TB often appears as acute, severe pneumonia. Nevertheless, characteristics that differentiate childhood TB from added origins of pneumonia are not deftly recognized.	Around 50% of pediatric-TB cases effectively reduce disease morbidity with preliminary empirical antimicrobial therapy. It has been interpreted as these cases frequently possess other microbial co-infection.	Clinicians must remember that diagnosing pediatric pneumonia and TB should be considered a possible cause, especially in high-burden settings. Those cases responded with traditional antimicrobials; appropriate diagnosis will be a problematic issue and follow-up.

MDR extrapulmonary tuberculosis (EPTB)

EPTB denotes TB involving organs except for the pulmonary tree [153]. The incidence of EPTB is higher in children than adults [154,155]. As EPTB affects various organs, clinical manifestations are vast and non-specific, making diagnosis difficult [156]. Drug resistance EPTB (DR-ERTB) cases also increased in the last decade [157]. A recent study has shown a 10-15% rise in DR-EPTB [157]. EPTB is less reported in children than in pulmonary tuberculosis (PTB) [154,158-160]. It has a higher incidence of MDR-TB [156, 161]. Case reports with MDR EPTB have been reported [162, 163], occur more in children under one year [164], and is associated with increased mortality [160]. X-pert Ultra is preferred as a diagnostic method for EPTB and drug resistance patterns [165,166]. Data on drug resistance in childhood EPTB is scarce [167]. EPTB profiles of children still need to be included in the WHO report [168].

## Conclusions

DR-TB is a potential threat to human health, especially to children. In this narrative review, it has been shown that under-reporting, lack of proper diagnostic tools, less accessibility to the health care system, and unavailability of suitable pediatric drugs against DR-TB, amalgamated with the COVID-19 pandemic have raised caseloads. Even then, negligence to this significant concern has been observed as there is a large gap in our knowledge of the epidemiology of pediatric DR-TB. Despite higher treatment success possibility in children, enrollment for treatment for DR is poorer in this age group, contributing to our future caseloads. WHO has incorporated all oral shorter regimens for children, but it is still not applicable for children of all ages. The performance of TB preventive therapy, as well as BCG (Bacillus Calmette-Guérin) vaccination coverage, is not satisfactory. Increased DR childhood TB means more potential future cases and is a significant obstacle to our SDGs of ending TB by 2030. In this dire condition, funding for TB has also been reduced, especially in low and middle-income countries (LMICs) where TB prevails most. National and International organizations should work diligently to combat the potential threat of the worldwide pediatric DR TB outbreak.
